# Note on the Origin of the Madagascar Strain of *Plasmodium vivax*

**DOI:** 10.4269/ajtmh.14-0507

**Published:** 2014-12-03

**Authors:** Andrew A. Lover

**Affiliations:** Infectious Diseases Programme, Saw Swee Hock School of Public Health, National University of Singapore, Singapore, E-mail: andrew.a.lover@gmail.com or ephaal@nus.edu.sg

Dear Sir:

Recent studies have established the potential for strains of *Plasmodium vivax* in Madagascar to infect Duffy-negative populations,^1^ and polymerase chain reaction (PCR)-based screening suggests a previously unrecognized burden of this parasite in sub-Saharan Africa.^2^

There is a dearth of data concerning any *P. vivax* parasites from Africa; however, one of the most widely studied strains used during the malariotherapy era was the Madagascar strain, used at the Horton Hospital research site in the UK from 1923 to ca. 1970. This isolate has been the subject of extensive studies,^3^,^4^ but there has been considerable debate about the geographic origin of this strain, and two recent papers specifically suggest the origin may well be elsewhere.^5^,^6^

However, further detail is provided in the work of Percy G. Shute, the clinician and malariologist who personally interviewed and treated the index patient for this strain. In his comprehensive report (which is not readily available) related to this clinical isolate, he states:

“The precise locality in Madagascar where the Indian seaman contracted the infection is impossible to determine. Ten days after his ship had left Madagascar, on the passage to England, he developed fever, but malaria was not diagnosed until he arrived at Tilbury (Port of London). He was then found to be suffering from a quotidian fever with temperatures rising to 40°–41°C. He gave no previous history of malaria and this attack was unlikely to have been a relapse for the reason that his paroxysms occured [sic] daily, as in a primary attack; whereas the fever in relapses is tertian from the start. For these reasons, it is most probable that the designation «Madagascar strain» is geographically correct.”^7^

These suggested differences in fever periodicity between primary infections and relapses are strongly supported by other clinicians in a range of settings,^8^,^9^ and this clinical behavior is likely a result of the slow establishment of well-synchronized parasite subpopulations within primary infections,^10^ in contrast to the emergence of clonal hypnozoites during relapses.^11^

These observations strongly suggest that the wealth of irreplaceable clinical and epidemiological data collected during malariotherapy treatments with the Madagascar strain have important and critical use, and should be combined with modern phylogenetic and molecular studies^12^,^13^ to inform a research agenda into the epidemiology of African *P. vivax*.
